# Advancing cortisol measurements in zebrafish: Analytical validation of a commercial ELISA kit for skin mucus cortisol analysis

**DOI:** 10.1016/j.mex.2024.102726

**Published:** 2024-04-18

**Authors:** Sara Jorge, Luís Félix, Benjamín Costas, Ana M. Valentim

**Affiliations:** a Instituto de Investigação e Inovação em Saúde, Universidade do Porto, (i3S), 4200-135 Porto, Portugal; b Centro Interdisciplinar de Investigação Marinha e Ambiental, (CIIMAR), 4450-208 Matosinhos, Portugal; c Instituto de Ciências Biomédicas Abel Salazar (ICBAS), Universidade do Porto, 4050-313 Porto, Portugal; d Centre for the Research and Technology of Agro-Environmental and Biological Sciences (CITAB), University of Trás-os-Montes and Alto Douro (UTAD), 5000-801 Vila Real, Portugal; e Instituto para a Inovação, Capacitação e Sustentabilidade da Produção Agroalimentar (Inov4Agro), University of Trás-os-Montes and Alto Douro (UTAD), Quinta de Prados, 5000-801 Vila Real, Portugal

**Keywords:** Zebrafish, Cortisol, Trunk, Skin mucus, ELISA, Validation, Skin mucus for cortisol measurement in zebrafish

## Abstract

Cortisol is the main stress biomarker used for zebrafish. However, zebrafish small size made it challenging to extract cortisol without harming or killing the fish. Thus, researchers adopted a terminal method, the trunk cortisol, as standard practice. Here, we developed and validated an alternative and minimally invasive technique for measuring cortisol in the skin mucus of adult zebrafish, using a commercial enzyme-linked immunosorbent assay (ELISA). For this, AB zebrafish were randomly assigned to a precision, accuracy, and specificity test. Each sample contained the skin mucus of five to ten fish or one fish trunk. The cortisol was extracted using methanol as organic solvent. The results obtained showed an adequate precision (intra-assay coefficient of variation (CV) <15%; inter-assay CV = 26%), accuracy (CV <120%), and specificity (r^2^ =0.96–0.98) for skin mucus cortisol levels, as well as for trunk cortisol.

• A commercial ELISA was analytically validated to measure cortisol in the skin mucus of zebrafish.
• Skin mucus cortisol is a non-terminal method that reduce the number of animals used and allows longitudinal studies.

## Graphical abstract

**Figure:**
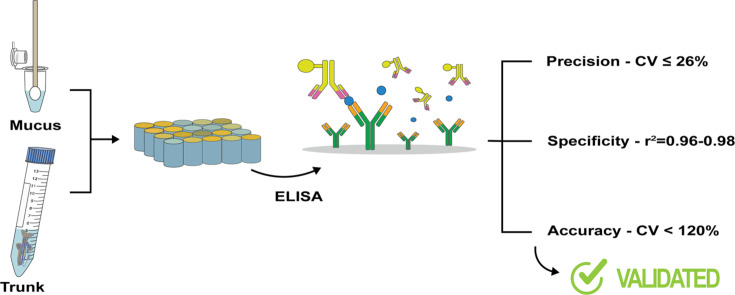

Specifications table

**Table:** 

Subject area:	Agricultural and Biological Sciences
More specific subject area:	*Animal Physiology and Endocrinology*
Name of your method:	*Skin mucus for cortisol measurement in zebrafish*
Name and reference of original method:	S. Jorge, L. Félix, B. Costas, A.M. Valentim, Housing conditions affect adult zebrafish (*Danio rerio*) behavior but not their physiological status, Animals 13 (6) (2023) 1120, https://doi.org/10.3390/ani13061120.
Resource availability:	*Materials and Reagents* 1. Sponges 2. Ophthalmologic scissor (Dentalhonest, Chengdu, China) 3. Clamp 4. Steal beads of 3 mm 5. Sterile swabs (Copan, Brescia, Italy) 6. ELISA kit (DetectX^Ⓡ^, Arbor Assays, Ann Arbor, Michigan; catalog number: K003-H1) 7. 2 mL Microtube Flat Cap (Deltalab SL, Barcelona, Spain) 8. Conical O-ring tubes 2 mL (Frilabo, Maia, Portugal) 9. Petri dishes (Fisher Scientific, Loughborough, United Kingdom) 10. Falcon 15 mL tubes (Enzymatic SA, Santo Antão do Tojal, Portugal) 11. Phosphate buffered saline,1x, pH 7.4 (Gibco™, Bio-Cult, Glasgow, Scotland) 12. Methanol (HPLC grade 99.8%; Fisher Scientific, Loughborough, United Kingdom) 13. n-Hexane (97+%; Acros Organics, Geel, Belgium) 14. 70% alcohol *Equipment* 1. Centrifuge 5415 R (Eppendorf, Hamburg, Germany) 2. FastPrep^Ⓡ^−24 (MP Biomedicals, Solon, Ohio, United States of America) 3. Freezer (−20 °C) 4. Fridge (4 °C) 5. Lab-roller (built in-house) 6. PowerWave XS2 microplate scanning spectrophotometer (Bio-Tek Instruments, Winooski, Vermont, United States of America) 7. Vacuum concentrator (Savant™ SPD131DDA SpeedVac™ Concentrator, ThermoScientific Inc., Waltham, Massachusetts, United States of America) 8. Vortex Mixer (Reagente 5 - Quimica E Electrónica, Lda, Porto, Portugal) *Software* 1. Gen5 data analysis software (BioTek Instruments Inc., Winooski, Vermont, United States of America) 2. IBM SPSS Statistics 27.0 computer program (SPSS, Chicago, Illinois, United States of America) 3. GraphPad Prism 6 for Windows (GraphPad, Inc., San Diego, California, United States of America)

## Method details

### Background

Zebrafish are increasingly being used as a stress model [1] due to the resemblance to humans endocrine-axis [2,3], sensibility to various stressors, complex behavioral repertoire [4], complete genome identification [5], and ease of manipulation under laboratory conditions [6].

To measure stress response, researchers typically rely on cortisol measurement. However, because of their small size, extracting cortisol from zebrafish can be challenging. Hence, cortisol is mainly extracted from their body [7]. Although this is a practical way to obtain cortisol, it is also terminal. Thus, the scientific community has been focusing on non-terminal alternatives such as housing water. Nonetheless, this approach has limitations too, including animals’ confinement to increase cortisol concentration in the water, and the complexity of sample processing, analysis, and data extraction (see [8]).

Skin mucus has recently been identified as a reliable non-terminal cortisol matrix for larger fish species such as seabream [9] and salmon [10], but has not yet been validated in zebrafish. In addition to being a natural barrier against stressors [9], its collection is minimally invasive and practical. Also, skin mucus production increases [11] and its composition changes when fish are stressed (e.g., [12,13]). Altogether, these characteristics make skin mucus a strong candidate to be studied in zebrafish as replacement for trunk cortisol.

Commercial Enzyme-Linked Immunosorbent Assays (ELISA) are a conventional method for cortisol measurement in zebrafish samples (e.g., trunk (e.g., [7,14]) or gill [15]) or housing water (e.g., [7,16]). These assays are advantageous since cortisol assessment may be completed quickly (within 4 h), without complex equipment [17] or training, and have excellent availability for an affordable price. However, they are mainly designed for human samples [18] and vary greatly regarding reagents and procedures [19]. Also, its increased usage was not accompanied by enough analytical validation [20].

Thus, the present study aimed to analytically validate a commercial ELISA kit for measuring cortisol in the skin mucus of adult zebrafish. We hypothesized that the chosen assay will precisely, accurately, and specifically detect cortisol in both the new (skin mucus) and standard (trunk) cortisol matrix.

### Animals and housing

Adults (less than 2-year-old) mixed-sex AB zebrafish were bred and housed in the i3S Animal facility. The animals were maintained in a thermoregulated recirculation water system, connected to a water purification unit, and equipped with 3.5 L tanks. The animals were subjected to a 14:10 h light: dark cycle and stable water quality with the following conditions: 27.0 ± 0.1 °C, pH of 7.4 ± 0.1, and conductivity of 900 µS. Fish were fed twice daily with a commercial diet (Zebrafeed 400–600 µm, Sparos, Olhão, Portugal) until apparent visual satiation.

### Experimental set-up

Each animal was randomly assigned to a specific cortisol matrix (skin mucus or trunk) and analytical validation test (precision, accuracy and specificity tests [21]). In the precision and accuracy tests, the skin mucus of five fish were pooled per sample (*n* = 5), whereas for trunk cortisol individual body samples of five fish were collected (*n* = 5). During the specificity test, to avoid cortisol signal loss due to sample dilution, each skin mucus sample (*n* = 5) was prepared by pooling the skin mucus of ten fish, whereas body samples of five fish were collected as one trunk per sample (*n* = 5).

### Sample collection

The animals for all tests were euthanized with rapid cooling (0–4 °C) at 10:00am, to prevent dial effect on cortisol levels [22]. To collect the mucus from the fish skin, the animals were first placed on a sponge soaked in cold water (0–4 °C). Then, their left side was swabbed with sterile swabs (Copan, Brescia, Italy) six times from the pectoral fins to the base of the caudal fin. In the middle of the third swabbing, the swab was rotated to collect as much mucus as possible. The left side of the fish was never in contact with the sponge to prevent any interference with the skin mucus collection. Five or ten fish's cotton tips, according to the test, were placed in a microcentrifuge tube with 750 or 2000 µL of ice-cold PBS (phosphate buffered saline, pH 7.4), respectively. For trunk sampling, the trunk samples were collected in 5000 µL of ice-cold PBS after decapitating the fish. If females, the eggs were removed, and the bodies cleaned in ice-cold PBS prior collection. The dissection tools were washed with ice-cold PBS and disinfected with 70% alcohol between animals. All samples were stored at −20 °C until processing for cortisol analysis.

### Sample preparation

1. Cut fish trunks with an ophthalmologic scissor (Dentalhonest, Chengdu, China) in 500 µL PBS.
2. Homogenize trunk samples by bead beating (five 3 mm steal beads) in the FastPrep^Ⓡ^−24 (MP Biomedicals, Solon, Ohio, United States of America; 6 m/s) for 60 s at room temperature.
3. Vortex skin mucus samples (Reagente 5 vortex mixer - Química E Electrónica, Lda., Porto, Portugal) for 2 min at a speed of 35 Hz.
4. Add to each trunk sample 750 µL of methanol (HPLC grade 99.8%; Fisher Scientific, Loughborough, United Kingdom).
5. Add 500 or 2000 µL methanol to each skin mucus samples with 5 or 10 fish's cotton tips, respectively.
6. Keep all samples (trunk and skin mucus) in a lab roller (built in-house according to Dhankani and Pearce [23]) at 60 rpm for 24 h at room temperature.
7. Centrifuge (Centrifuge 5415 R, Eppendorf, Hamburg, Germany) samples at 10,000 g for 10 min at 4 °C.
8. Discard the swabs of the skin mucus samples and transfer the supernatant of all samples (trunk and skin mucus) to new microcentrifuge tubes.
9. Evaporate tubes at 36 °C on a vacuum concentrator (Savant™ SPD131DDA SpeedVac™ Concentrator, ThermoScientific Inc., Waltham, Massachusetts, United States of America).
10. Reconstitute cortisol volume of the trunk samples with 500 µL PBS.
11. Reconstitute the volume of skin mucus samples according to the assay test: 125 µL for the specificity test and 260 µL for the precision and accuracy test.
12. Keep all samples overnight at 4 °C.
13. Add 500 µL n-Hexane (97+%; Acros Organics, Geel, Belgium) to trunk samples to remove the interference of precipitated lipids. After 15 min of freezing at −20 °C, discard the organic layer of these samples.

### Sample analysis

Prior to the method validation, kits from various brands were tested, including one previously used by our team (see [24]). The DetectX^Ⓡ^ kit (Arbor Assays, Anne Arbor, MI; K003-H1) outperformed the other kits and was selected for further analysis. The cross-reactivity of this kit antibody to other steroids is 18.8% for the dexamethasone, 7.8% for the prednisolone (1-dehydrocortisol), 1.2% for the corticosterone and the cortisone, according to the manufacturer. Cross-reactivity for progesterone, estradiol, cortisol 21-glucuronide, 1α-hydroxycorticosterone and testosterone is less than 0.1%. According to this, we do not expect a problem of cross-reactivity with our zebrafish samples.

The cortisol analysis was performed according to the manufacturer instructions, with minor alterations:

1. Add 25 µL of standards or samples to the microplate wells in duplicate.
2. Add 25 µL of cortisol conjugate and antibody to the microplate wells.
3. Tap plate three times.
4. Incubate plate in the dark for 1 h at room temperature.
5. Discard the liquid and add 200 µL of wash buffer to the microplate wells.
6. Repeat the previous step three times, with the plate being tapped in absorbent paper after each wash.
7. Add 100 µL of 3,3′,5,5′-tetramethylbenzidine substrate solution to each well.
8. Incubate the plate for 30 min at room temperature.
9. Add 50 µL stop solution to each well.
10. Read the plate at 450 and 490 nm at 25 °C using a PowerWave XS2 microplate scanning spectrophotometer (Bio-Tek Instruments, Winooski, Vermont, United States of America) with Gen5 data analysis software (BioTek Instruments Inc., Winooski, Vermont, United States of America).

The bounded cortisol was calculated through the division of the mean of absorbance from the duplicates for each sample or the absorbance of the kit standard solutions (B) by the absorbance of the well without added cortisol (B0). Cortisol concentrations are presented as nanograms per deciliter.

## Method validation

The DetectX^Ⓡ^ assay was validated using fundamental criteria for analytical validation: precision, specificity, accuracy, and sensitivity [21]. However, the sensitivity, which is the lowest level of cortisol that the assay kit is capable of detecting, was not calculated, as the manufacturer had already estimated it to be 0.00276 µg/dL.

### Precision

The assay precision was determined by the calculation of an intra-assay and inter-assay coefficient of variation (CV).

The intra-assay for skin mucus (CV=14%) and trunk samples (CV=5%) was within the acceptable range (up to 20%, as indicated by [25,26]). The CV was calculated using the cortisol levels measured in each duplicated sample (*n* = 5) and using the formula:

$$\text{CV}=\;\frac{\text{standard}\;\text{deviation}\;(\mu g/\text{dL})\;}{\text{mean}\;(\mu g/\text{dL})}\;x\;100$$

To our knowledge, only one study [7] tried to validate the Detect X assay to quantify cortisol in zebrafish trunk, but they only reported intra-assay CV (7.75%). We cannot compare this value with ours (5%), because the CV reported was a mean of the cortisol CV from the trunk and water samples.

For the inter-assay the mean of the cortisol levels from the duplicated samples in each plate was used in the formula:

$$\text{CV}=\;\frac{\text{standard}\;\text{deviation}\;\text{of}\;\text{three}\;\text{plates}\;(\mu g/\text{dL})\;}{\text{mean}\;\text{of}\;\text{three}\;\text{plates}\;(\mu g/\text{dL})}\;x\;100$$

However, one mucus sample was excluded from the inter-assay analysis, since a duplicate plate's absorbance was unreadable in two of the three plates. The inter-assay CV obtained for both cortisol matrices were 26%, slightly exceeding the upper limit of 20% that is commonly reported (e.g., [25,26]). Nevertheless, given that the samples in our study contained reduced amounts of biological material, the obtained results can still be regarded as valid. This is in line with recent European Union guidelines [27], which acknowledge CVs of up to 30% in such cases.

### Specificity

The specificity was assessed through a linearity test, assuming that, by serially diluting the matrices samples with PBS, the diluted samples would be parallel to the cortisol standard curve of the assay. The dilution ratios utilized were 1:2, 1:3, and 1:4. The specificity of the assay for each dilution was also calculated, considering the formulas:

$$\text{Expected}\;\text{cortisol}\;\left(\frac{\mu g}{\text{dL}}\right)=\;\frac{\text{measured}\;\text{cortisol}\;\text{of}\;\text{original}\;\text{sample}\;(\mu g/\text{dL})\;}{\text{dilution}\;\text{denominator}}$$

And,

$$\text{Recovery}\;\left(\%\right)=\frac{\text{measured}\;\text{cortisol}\;\left(\frac{\mu g}{\text{dL}}\right)}{\text{expected}\;\text{cortisol}\;\left(\frac{\mu g}{\text{dL}}\right)}x\;100$$

**Fig. 1:**
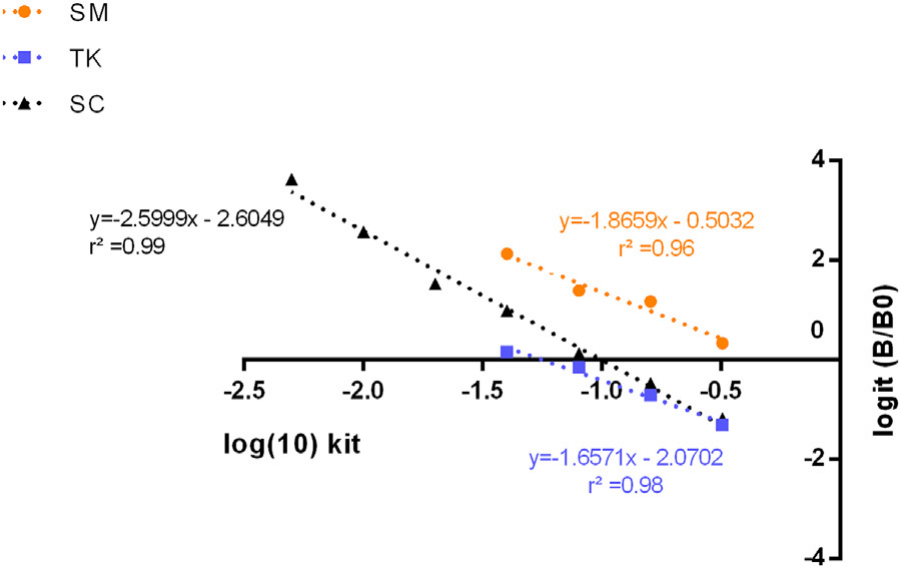
Parallelism response between the ELISA standards (SC) and the serial dilutions (1, 1:2, 1:3 and 1:4) of skin mucus (SM) and trunk (TK) cortisol samples; B- absorbance of the sample; B0- absorbance of the well without cortisol. The samples (*n* = 5) and seven standards were run in duplicates over a period of one week in two independent assays. Each point is the mean of samples and standards read in duplicate. More details can be found in Fig. S1.

The difference between the measured and expected cortisol levels was also assessed through a repeated measures ANOVA using cortisol (measured vs. expected) as between-subjects factor and dilution rate as within-subjects factor. For this, data was checked for normality and homogeneity by the Shapiro-Wilk and Levene's tests, respectively. The data analysis was done using the IBM SPSS Statistics 27.0 computer program (SPSS, Chicago, Illinois, United States of America) and considering a difference significant at *p* < 0.05. The sigmoidal curve from the ELISA kit and the cortisol data were log-transformed before being plotted to evaluate parallelism using GraphPad Prism 7 for Windows (GraphPad, Inc., San Diego, California, United States of America). Diluted skin mucus and trunk samples showed a parallel response with the assay cortisol standards (Fig. 1). The lines had a slope of −2.60 for the assay standards and −1.66 and −1.87 for the trunk and skin mucus samples with r^2^ values of 0.98 and 0.96, respectively. The repeated measures ANOVA showed no difference between the measured and expected skin mucus (F(1,8)=0.248, *p* = 0.632) and trunk (F (1,8) =0.070, *p* = 0.798) cortisol levels, corroborating an adequate assay specificity. Skin mucus (F(2,16)=58.110, *p* < 0.001) and trunk (F(2,9.468)=12.691, *p* = 0.004) cortisol samples showed differences in dilutions as expected. There was no interaction between dilution and cortisol factors either for skin mucus (F(2,16) =2.057, *p* = 0.160) or trunk (F(1,8)=0.437, *p* = 0.527). The mean recovery rates of cortisol obtained for skin mucus (108 ± 5.6%) and trunk (101 ± 9.4%) cortisol were acceptable for validation.

### Accuracy

The accuracy of the assay was evaluated using a spike and recovery test, after adding known cortisol concentrations (standards 0.32, 0.16 and 0.08 µg /dl) to the samples. The standards and samples were mixed 1.1 (v:v). The accuracy data is expressed as % recovery after applying the formula:

$$\text{Recovery}\;(\%)=\;\frac{\text{Measured}\;\text{cortisol}\;(\mu g/\text{dL})\;}{\text{Expected}\;\text{cortisol}\;(\mu g/\text{dL})}\;x\;100$$

Where,

$$\text{Expected}\;\text{cortisol}\;(\mu g/\text{dL})=\;\frac{\text{Measured}\;\text{cortisol}\;\text{of}\;\text{original}\;\text{sample}\;(\mu g/\text{dL})\;+\;\text{added}\;\text{concentration}\;(\mu g/\text{dL})\;}{2}$$

The mean % of skin mucus and trunk cortisol recovered from spiked cortisol samples (Table 1) were 107 and 92%, respectively. These values cover the acceptable range of accuracy (80–120%) for ELISA validation (see [25]).

**Table 1:** Mean percentage of trunk and skin mucus (*n* = 5) cortisol recovered after the accuracy test. Data are expressed as mean ± standard deviation.

Matrix	Added concentration to X (µg/dL)	Measured cortisol (µg/dL)	Expected cortisol (µg/dL)	Recovery (%)
Trunk	X	0.23 ± 0.16	0.23 ± 0.16	100
	0.32	0.25 ± 0.08	0.28 ± 0.08	91
	0.16	0.18 ± 0.07	0.20 ± 0.08	94
	0.08	0.14 ± 0.08	0.16 ± 0.08	91
				
Skin mucus	X	0.02 ± 0.01	0.02 ± 0.01	100
	0.32	0.19 ± 0.04	0.17 ± 0.01	108
	0.16	0.09 ± 0.005	0.09 ± 0.01	102
	0.08	0.06 ± 0.01	0.05 ± 0.01	112

X = Cortisol value in µg/dL of the original sample.

## Conclusions

Overall, the results obtained validate the DetectX^Ⓡ^ assay as a reliable ELISA kit for cortisol measurement in the skin mucus and trunk of adult zebrafish. Even with half of the recommended sample volume (25 µL), the criteria for analytical validation were covered.

Also, this study proved the suitability of skin mucus to be used as a replacement of trunk matrix for cortisol measurement in zebrafish. The use of skin mucus offers a non-terminal, practical and time-efficient approach to cortisol analysis in zebrafish, with a wide range of applications (e.g., health and environmental monitoring; study of stress models).

## CRediT authorship contribution statement

**Sara Jorge:** Validation, Investigation, Formal analysis, Writing – original draft, Methodology, Conceptualization, Writing – review & editing. **Luís Félix:** Visualization, Conceptualization, Writing – review & editing. **Benjamín Costas:** Resources, Conceptualization, Writing – review & editing. **Ana M. Valentim:** Supervision, Conceptualization, Writing – review & editing.

## Declaration of competing interest

The authors declare that they have no known competing financial interests or personal relationships that could have appeared to influence the work reported in this paper.

## Data Availability

Data will be made available on request.

## Appendix Supplementary materials

Image, application 1
