# Supplementary material for: Advancing cortisol measurements in zebrafish: Analytical validation of a commercial ELISA kit for skin mucus cortisol analysis
Source: MethodsX. 2024 Apr 18;12:102726. doi: 10.1016/j.mex.2024.102726 (PMC11068847; doi:10.1016/j.mex.2024.102726)

**Fig. S1. Mean ± standard deviation of the parallelism response.**

Parallelism response between the seven ELISA standards (SC) and the serial dilutions (1, 1:2, 1:3 and 1:4) of skin mucus (SM) and trunk (TK) cortisol samples (n=5); B: mean absorbance; B0: blank values. Data are expressed as mean ± standard deviation.


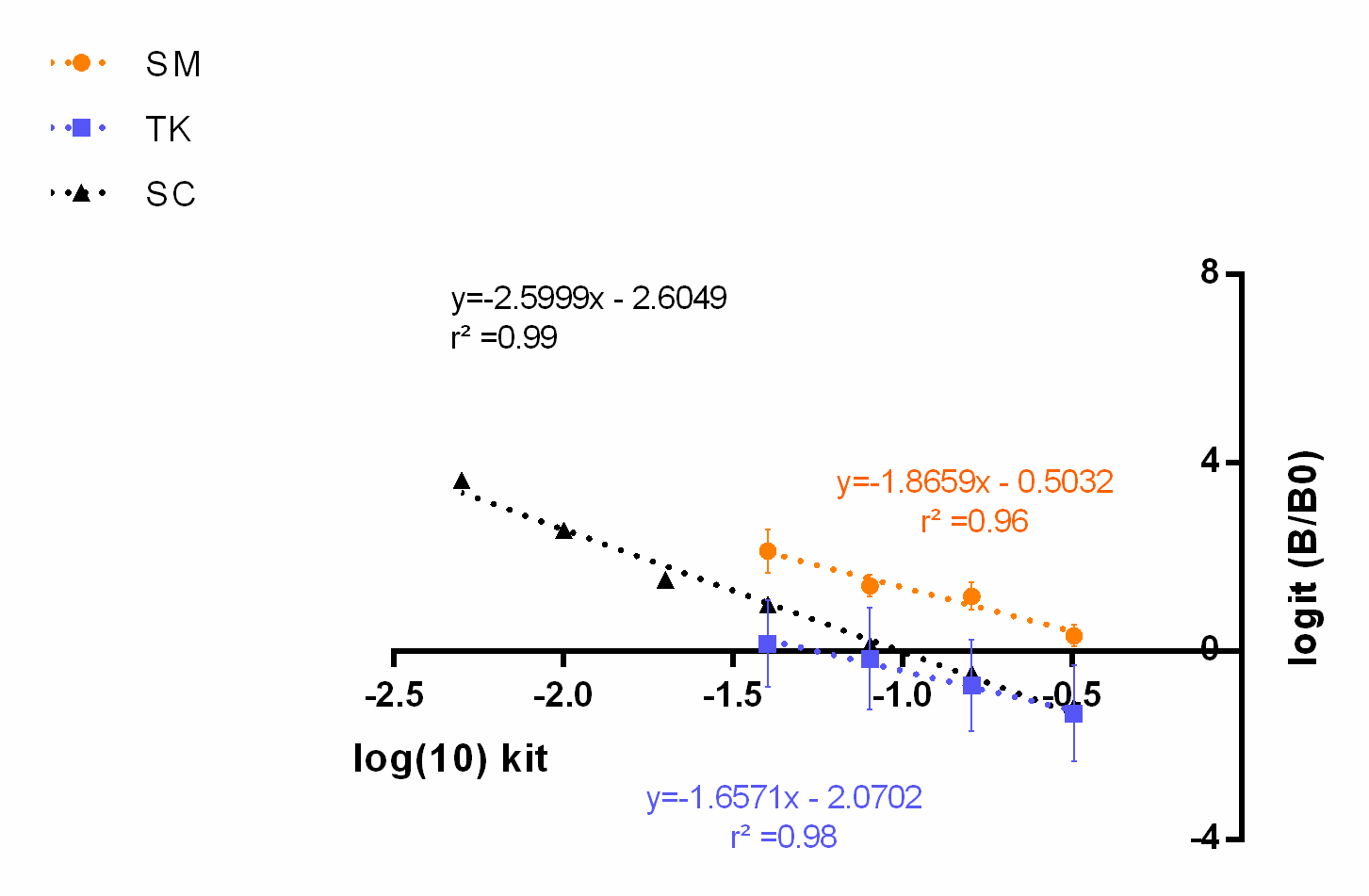

Supplement: Supplementary file 1 [file mmc1.docx]
